# Does SLC17A9 (VNUT) function as a stand-alone nucleotide and/or phosphate transporter?

**DOI:** 10.1016/j.jbc.2026.113360

**Published:** 2026-07-22

**Authors:** Jennifer Struck, Vivek Malviya, Marcelo Ganzella, Abhishek Acharya, Brigitte Barg-Kues, Sivakumar Sambandan, Gudrun Ahnert-Hilger, Reinhard Jahn

**Affiliations:** Laboratory of Neurobiology, Max-Planck-Institute for Multidisciplinary Sciences, Göttingen, Germany

**Keywords:** vesicular neurotransmitter transporter, functional reconstitution, vesicular glutamate transporter, electrochemical proton gradient, Na^+^-dependent phosphate transport, synaptic vesicles

## Abstract

The solute carrier SLC17A9 was previously identified as a vesicular nucleotide transporter that is primarily responsible for transporting ATP and related nucleotides into intracellular vesicles, and its deletion in mice resulted in a loss of ATP from intracellular storage organelles. Using recombinant SLC17A9 and functional reconstitution in liposomes we have now attempted to characterize the molecular features of its transport activity. While the purified protein includes the entire open reading frame, forms dimers, and is apparently properly folded and correctly oriented in the liposomes, we were unable to measure any nucleotide transport activity although the electrochemical potential was intact, the liposomes were not leaky, and the related vesicular glutamate transporter (SLC17A57) reconstituted in parallel under the same conditions was active. We also tested whether SLC17A9, similar to related transporters in the SLC17 family, functions as a Na^+^-dependent phosphate transporter. However, no such activity was detectable, and expression of the transporter in HeLa cells did not result in consistent increases of phosphate uptake. We conclude that the role of SLC17A9 in vesicular nucleotide transport needs further corroboration, possibly requiring additional, hitherto unknown factors.

Adenosine triphosphate (ATP) not only serves as energy donor in countless metabolic reactions but also functions as an extracellular signaling molecule that operates by activating purinergic receptors (1, 2). While ATP can be released directly from the cytoplasm *via* nonselective channels such as pannexins or calcium homeostasis modulator 1 (3, 4, 5, 6), a major route for ATP release is by exocytosis from intracellular storage vesicles. Indeed, secretory organelles from diverse tissues such as neuronal synaptic vesicles, adrenal chromaffin granules, insulin-containing granules, or secretory lysosomes from various cell types are known to contain ATP, in some cases at very high (hundreds of mM) concentrations (reviewed by (7, 8, 9)). In many secretory vesicles, ATP is co-stored with other neurotransmitters such as aminergic transmitters or acetylcholine (summarized in (7, 9), and co-release was also reported with GABA (8, 10).

Since many years it is known that secretory organelles sequester ATP by active transport. Organellar uptake of ATP depends on an electrochemical proton gradient (ΔμH^+^) that is generated by a vacuolar proton ATPase (v-ATPase) present on all post-Golgi trafficking vesicles. In 2008, Sawada and colleagues identified a member of the solute carrier superfamily, SLC17A9, as a transporter responsible for ATP uptake, henceforth also termed vesicular nucleotide transporter (VNUT) (11). Using *in vitro* reconstitution of purified transporter protein, the authors showed that vesicular uptake of ATP can be induced by creating an artificial membrane potential (Δψ, inside positive) across the liposome membrane. Further characterization revealed similarities with the vesicular neurotransmitter transporters, particularly the glutamate transporters VGLUTs 1-3 (SLC17A6-8) that are structurally related and belong to the same subgroup of solute carriers (12, 13). The similarities include a dependence on the membrane potential rather than the proton gradient ΔpH, and a strong activation of transport by mM concentrations of chloride (Cl^-^)ions (11, 14). Like VGLUTs, SLC17A9 is inhibited by azo dyes such as Evans Blue, ketone bodies, and the stilbene anion channel blocker DIDS. In addition, the bisphosphonate clodronate (licensed in some countries as an anti-osteoporotic drug) was shown to be specific and highly potent in inhibiting SLC17A9 activity, possibly by interfering with Cl^-^-binding (15). Based on what we know so far, these features resemble those of ATP uptake by purified secretory vesicles, in particular chromaffin granules (16, 17).

Support for the identity of SLC17A9 as *bona fide* ATP-transporter was provided by an analysis of transgenic KO mice in which expression of the SLC17A9 protein was prevented (18). Surprisingly, the mice were healthy and did not exhibit obvious developmental, behavioral, or metabolic phenotypes (note that in a more recent study, conditional KO of SLC17A9 in astrocytes has been shown to result in behavioral abnormalities such as increased anxiety and depressive-like features (19)). However, in the KO mice ATP-release was abolished from chromaffin cells as well as from pancreatic beta cells, although exocytosis was not impaired, with insulin release rather being enhanced. Further analysis revealed that ATP storage and release was impaired from many other cell types such as platelets (20), neutrophils (21), microglia, and astrocytes (22); for review, see (8). In adrenal chromaffin cells, knockdown of SCL17A9 and the resulting reduction of granular ATP content resulted in a massive reduction of adrenalin storage (23), supporting the view that catecholamines need to be co-concentrated with ATP for reaching hyperosmolar concentrations in the vesicles.

Biochemical reconstitution of purified SLC17A9 revealed a rather broad substrate specificity, including ATP, GTP, UTP, ITP, ADP, and diadenosine triphosphate as substrates. AMP also appears to be transported, albeit with lower affinity, whereas other neurotransmitters such as glutamate are not recognized (14, 15, 20, 24); for review, see (25). Intriguingly, and in contrast to other ATP-handling transporters or exchangers, ATP transport is not affected regardless of whether Mg^2+^ is present or not, but when present at mM concentrations, Mg^2+^ or Ca^2+^ appear to be co-transported with ATP (14).

Motivated by these results, we set out to characterize the biochemical and biophysical properties of SLC17A9 further in order to shed more light on its transport mechanism, with particular emphasis on similarities and differences to the structurally related VGLUT transporters. Moreover, we asked whether SLC17A9 also functions as a sodium-dependent transporter of inorganic phosphate ions (P_i_), a feature that appears to be shared by most other SLC17A family members.

## Results

### Expression, purification, and initial characterization

**Figure 1:**
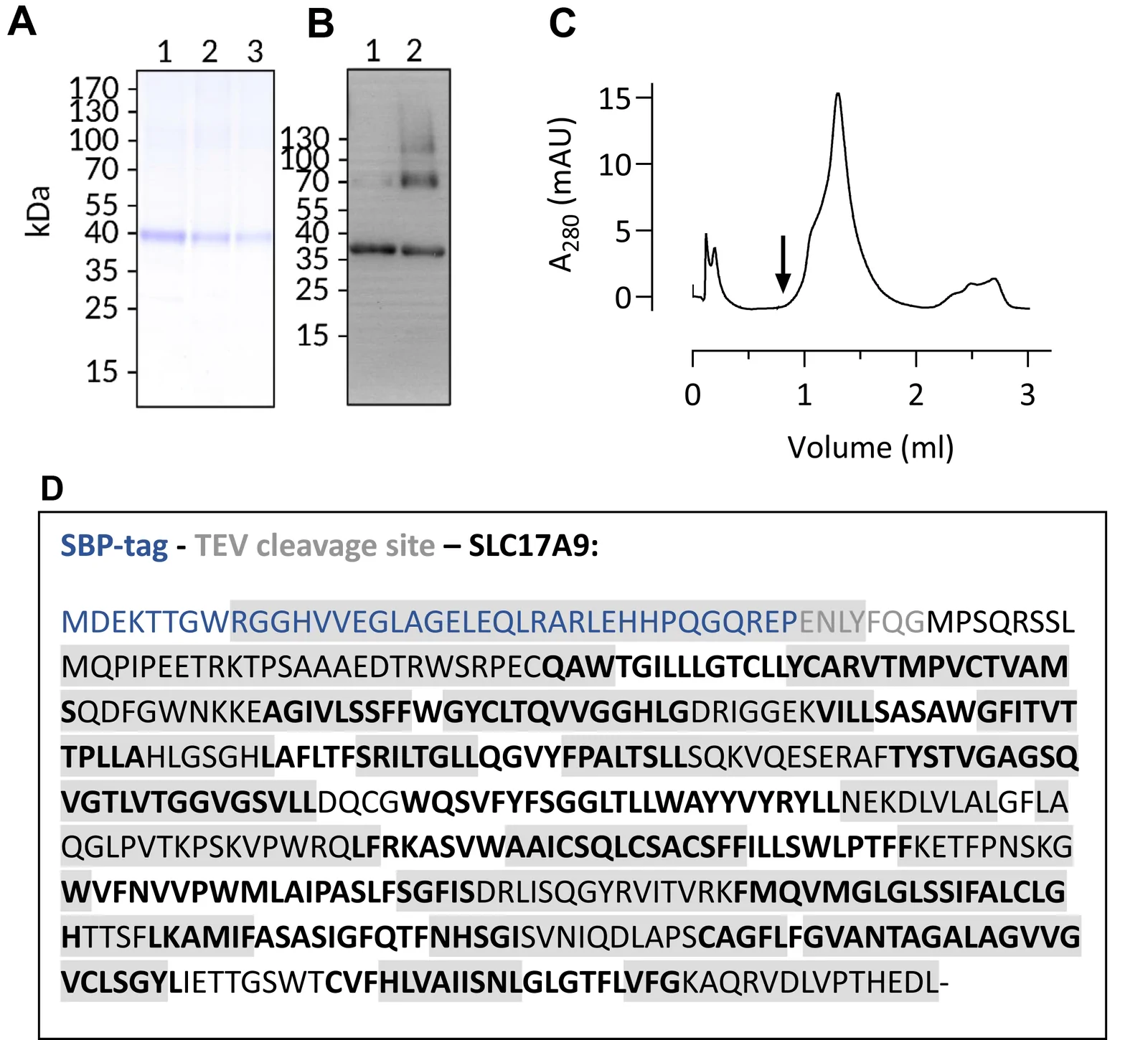
**Characterization of purified recombinant SLC17A9.***A*, separation by SDS-PAGE of elution fractions derived from the affinity column, showing the purified SBP-tagged SLC17A9 protein. 30 μl of elution sample was loaded in each lane and 1, 2 and 3 lanes contain samples from three subsequent elutions of 1.5 ml each. The protein, visualized by Coomassie *Blue* staining, migrates as a single major band with an apparent Mr of 40 kDa. *B*, immunoblot of two purified preparations of recombinant SLC17A9 protein containing different affinity tags. Lane 1, SLC17A9 containing Twin-Strep-Tag at the C-terminus; lane 2, SLC17A9 containing an N-terminal SBP-tag (as in *A*). 1 μg of protein/lane was analyzed, and SLC17A9 was detected using a SLC17A9-specific antibody. *C*, elution profile of purified SLC17A9 protein (in DDM) from a size-exclusion column (see text for details). The *arrow indicates* the elution of the excluded void volume. Purified SLC17A9 eluted as a symmetrical peak with a shoulder, probably representing a monomer-dimer equilibrium (see text). *D*, amino acid sequence corresponding to the open reading frame of the cDNA construct used for protein expression, including the N-terminal SBP-tag that is connected to the full-length sequence of SLC17A9 by a short linker containing cleavage site for TEV protease. The purified protein was analyzed by LC-MS/MS, with the recovered peptides being shaded.

Full-length SLC17A9 (mouse), tagged N-terminally with a streptavidin-binding peptide (SBP) that was connected to the protein by a linker containing a cleavage site for TEV protease, was expressed in insect cells using the baculovirus system. After cell disruption and the isolation of a membrane fraction by differential centrifugation, proteins were solubilized in the detergent dodecylmaltoside (DDM, 2% w/v), followed by affinity adsorption on a column containing streptavidin beads. The column was then washed (allowing for buffer and detergent exchange), and the protein was eluted using biotin. After cleaving off the SBP-tag, analysis by SDS-PAGE revealed a single major band migrating at an apparent molecular mass of 40 kDa (Fig. 1*A*).

While the apparent Mr of our purified protein as determined by SDS-PAGE is only slightly below the predicted Mr of 48,470 Da (mouse), it is considerably lower than that reported in previous studies (ranging between 60 and 80 kDa (11, 20, 21, 23, 26)). For this reason, we carried out several control experiments to ensure that the expressed and purified protein encompasses the entire open reading frame of SLC17A9. First, we expressed and purified a second variant of SLC17A9 that contains another tag (Twin-Strep-tag) at the C-terminus and then checked by immunoblotting whether the apparent Mr is dependent on whether the affinity tag is added either to the N-terminus (SBP-tag) or to the C-terminus (Twin-Strep-tag). This was not the case (Fig. 1*B*). The purified SBP-tagged protein was also examined by size-exclusion chromatography (in DDM) (Fig. 1*C*). The protein eluted as a major peak with a shoulder, both of which contained the 40 kDa band as a single major component, with the two components possibly being caused by a monomer–dimer equilibrium (see below).

Second, the purified protein containing the N-terminal SBP-tag was analyzed by mass spectrometry, resulting in a coverage of most of the open reading frame, including peptides encompassing the predicted N- and C-terminal regions (Fig. 1*D*). Also, note that upon expression in HeLa cells, the protein migrated around 40 kDa (see below). Taken together, these data confirm that the expressed protein corresponds to the full-length open reading frame and is not truncated or partially degraded.

**Figure 2:**
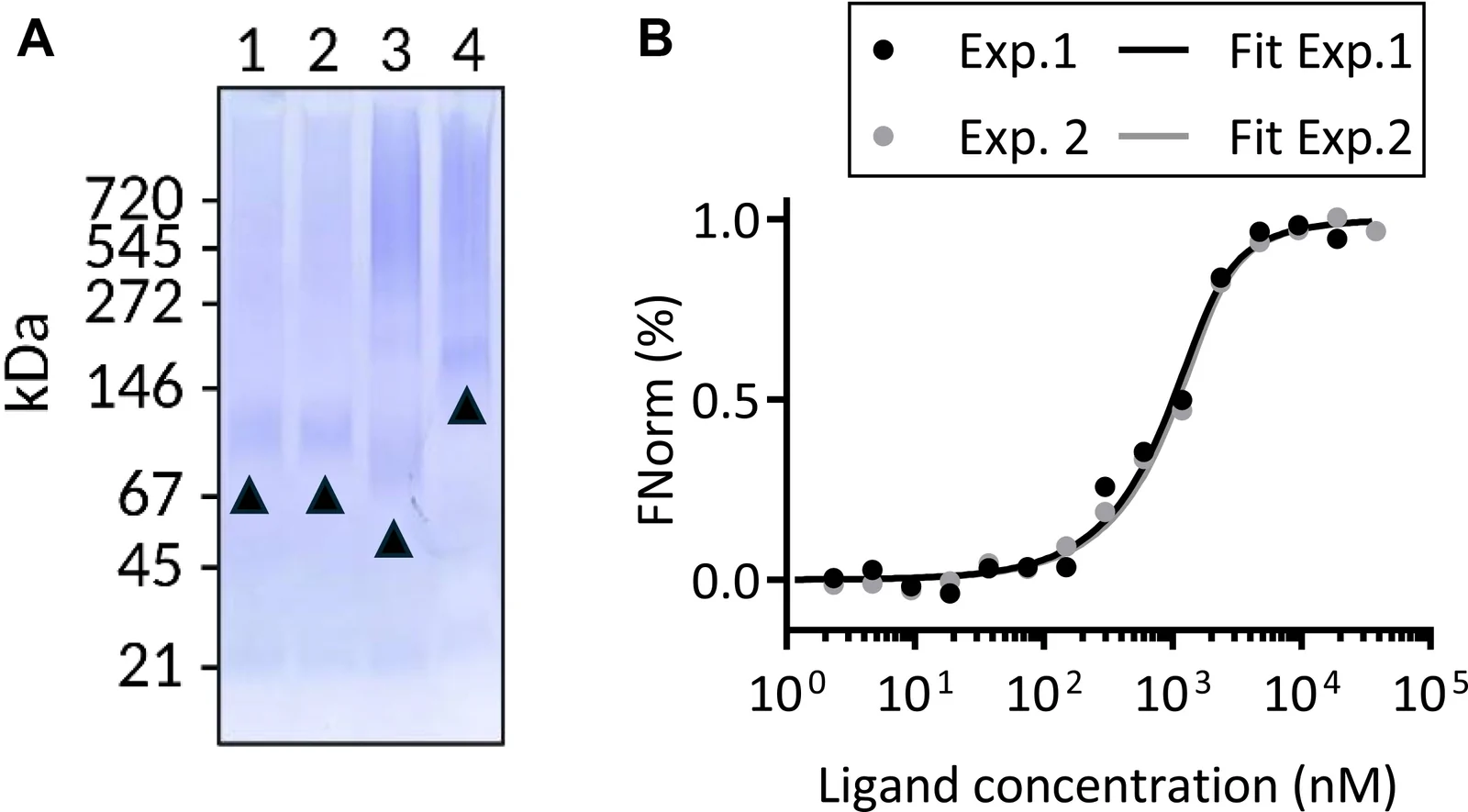
**SLC17A9 forms dimers and possibly also higher oligomers.***A*, separation of SLC17A9 protein, purified in different detergents, using *Blue* Native polyacrylamide gel electrophoresis. Each lane contains 30 μg of protein and 0.025% Coomassie *Blue* G-250 dye. Lane 1: dodecylmaltoside (DDM); lane 2: lauryl maltose neopentyl (LMNG); lane 3: amphipole A8-35; lane 4: glyco-diosgenin (GDN). Depending on the type of detergent, the protein (*arrows*) migrates at different positions, with the apparent Mr corresponding to dimers or even higher oligomers. *B*, association of non-labeled SLC17A9 with labeled SLC17A9, measured by microscale thermophoresis (MST). In this experiment, 1 nM of protein labeled by Alexa-fluor 647 was titrated increasing amounts of non-labeled protein (highest concentration 38 μM). After loading in capillaries, each sample was heated by illumination at 650 nm, followed by measuring thermophoresis of the fluorescent protein. The data were then plotted to generate a binding curve, yielding an apparent K_D_ of ∼12 μM.

**Figure 3:**
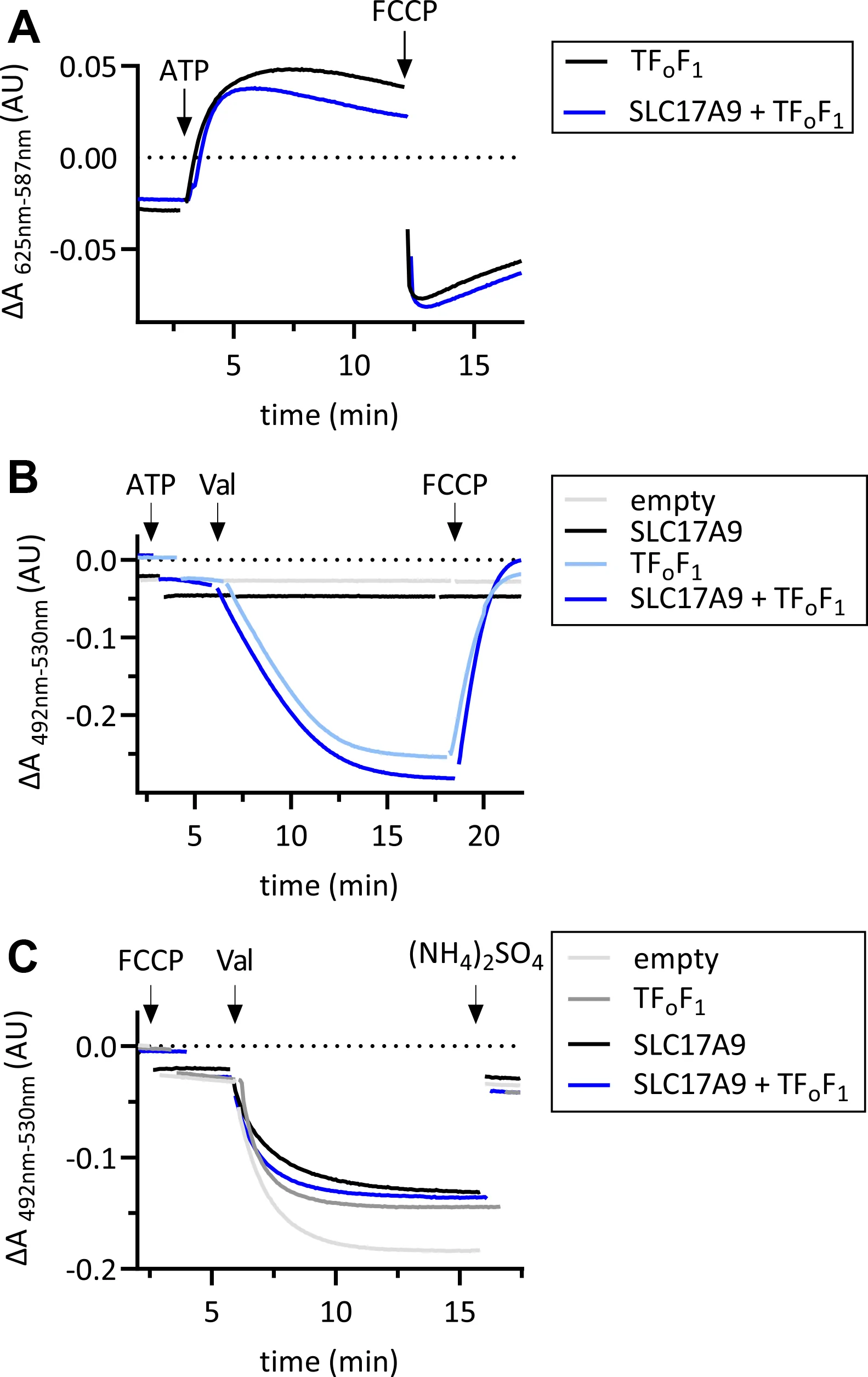
**SLC17A9-containing proteoliposomes establish stable electrochemical proton gradients.** Proteoliposomes reconstituted with purified SLC17A9 protein, and/or the proton ATPase TF_o_F_1_ establish stable membrane potentials (Δψ, inside positive), or pH-gradients (ΔpH, inside acidic) under appropriate conditions. The panel show absorption changes of reporter dyes (oxonol VI for Δψ (*A*), and acridine orange for ΔpH (*B*, *C*)), recorded in the dual wavelength mode (see methods). *A*, addition of ATP leads to the development of an inside positive membrane potential, that is dissipated by the protonophore FCCP. *B*, addition of ATP does not create a major pH-gradient unless charge compensation is enabled by K^+^-efflux, induced by the K^+^-ionophore valinomycin (Val). *C*, in the absence of TF_o_F_1_, a stable pH-gradient is created using an imposed K^+^-diffusion potential. Liposomes preloaded with 150 mM K^+^ are incubated in K^+^-free buffer, followed by selective permeabilization of the membrane for protons (FCCP) and K^+^-ions (Val). Note that in *A*, standard liposomes with the Twin-Strep-tagged SLC17A9 were used whereas in *B* and *C* SBP-tagged SLC17A9 protein was used. Moreover, the liposomes used in *B* and *C* were preloaded with the ATP-sensor ATeam (see text).

**Figure 4:**
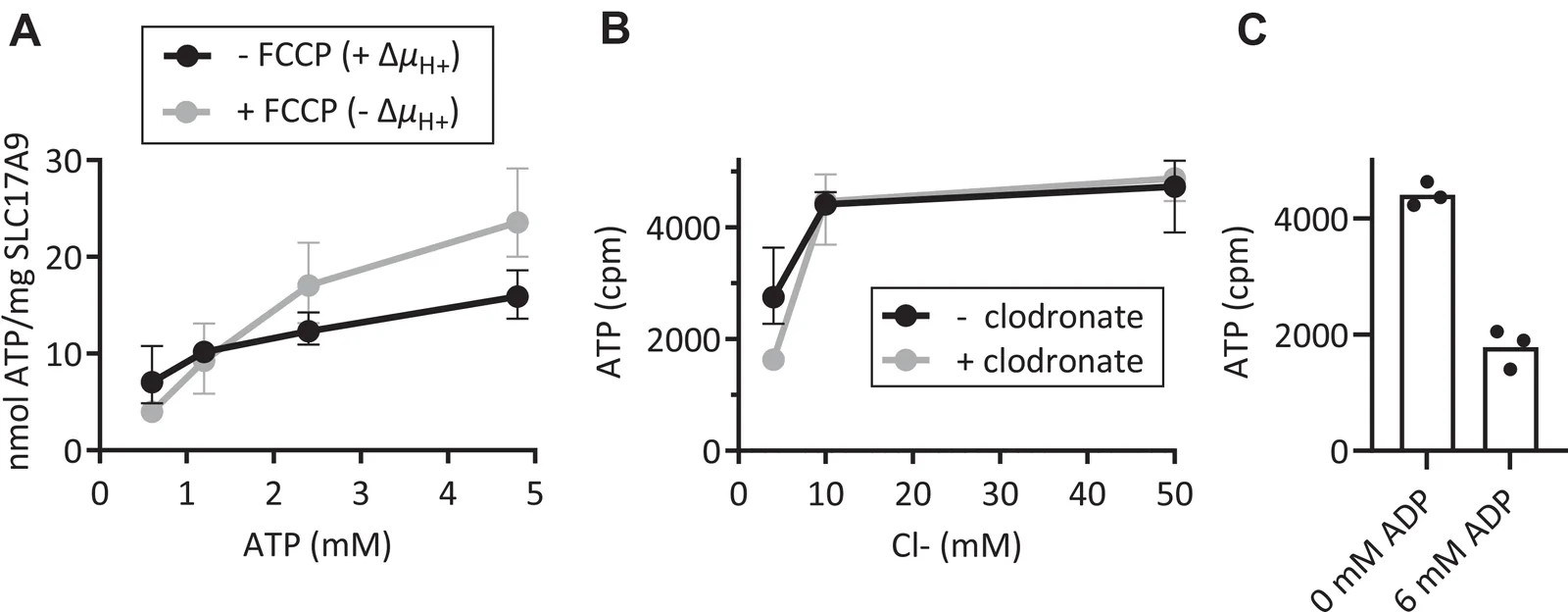
**SLC17A9-containing proteoliposomes do not transport ATP when electrochemical proton gradients are applied.** Absence of proton-gradient-dependent or clodronate-sensitive uptake of ATP by proteoliposomes containing Twin-Strep tagged SLC17A9 and TF_o_F_1_ under conditions maximizing Δψ (see Fig. 3*A*). The figure shows examples of many similar experiments with varying experimental conditions. The figure shows data from three experiments (in *A* and *B* the bars show the range between the lowest and highest data point). *A*, ATP uptake/binding in dependence on increasing ATP concentrations. *B*, ATP uptake/binding (0.5 mM ATP) at increasing chloride concentrations. *C*, competition of the clodronate-insensitive increase at 10 mM Cl^-^ (that is also largely insensitive to blockers of the electrochemical gradient, not shown) by excess of ADP. Similar results were obtained in the presence of excess unlabeled ATP (not shown).

**Figure 5:**
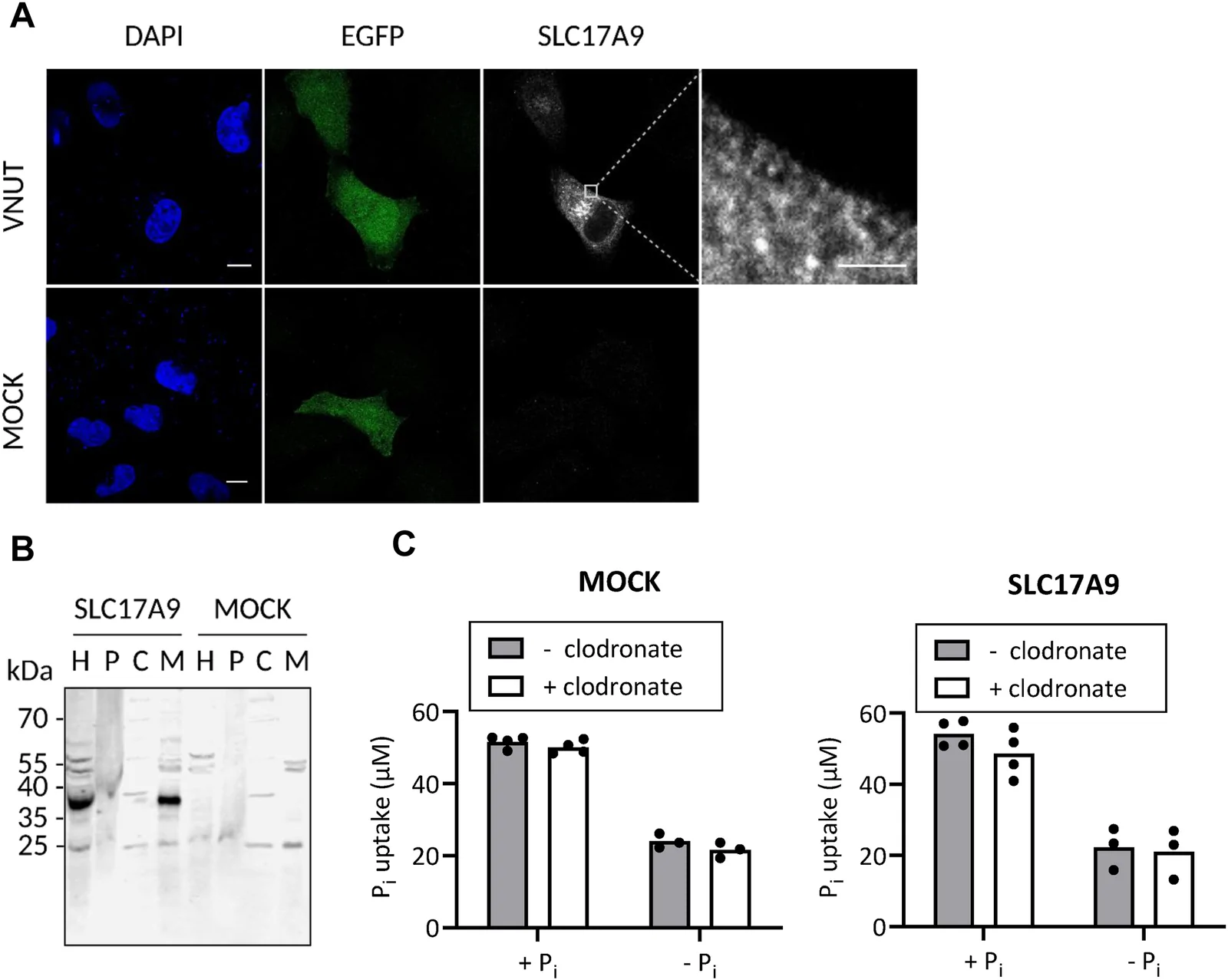
**No clear evidence for SLC17A9-dependent Pi-transport by HeLa cells expressing the protein.***A*, fluorescence micrographs showing HeLa cells transiently transfected with SLC17A9 and eGFP (*top*) or eGFP alone (MOCK, *bottom*). *Blue*: DAPI staining for showing nuclei of all cells, *Green*, eGFP-fluorescence, *white*: immunostaining for SLC17A9. Scale bar: 10 μm, inset 2.5 μm. *B*, expression of SLC17A9 in a membrane fraction obtained from HeLa cells, detected by immunoblotting after separation by SDS-PAGE. HeLa cells were transiently transfected as in *A*, followed by homogenization (yielding homogenate H), and 2-step differential centrifugation, yielding a low-speed pellet (P), a high-speed membrane pellet (M), and a soluble fraction (C). Protein expression is detected in the homogenate and in the membrane fraction of cells transfected with SLC17A9 but not in control (eGFP) transfections. C: Uptake of P_i_ by HeLa cells expressing SLC17A9. After preincubation for 1 h in P_i_-free buffer, cells were re-incubated for 15 min in KREBS buffer containing 10 mM P_i_. Where indicated, 100 μM of clodronate were added. The cells were then harvested, and the P_i_-content was determined (see Experimental procedures for details). Data points of 3 to 4 independent experiments are shown.

Considering the discrepancies in the reported Mr, we investigated whether solubilized SLC17A9 may tend to form oligomers Indeed, when analyzed by blue native electrophoresis, a non-denaturing method known to preserve complexes of membrane proteins (27), the protein migrated with apparent molecular masses between 80 and 200 kDa, depending on the detergent used, indicative of dimer or even higher oligomeric forms (Fig. 2*A*, see also Fig. S3*A*). Thermal denaturation experiments revealed that the protein remains properly folded in all tested surfactants except amphipole, yielding clear unfolding transitions between 39.7 °C (DDM) and 70.5 °C (GDN) (Fig. S1).

To further analyze whether the purified transporter forms monomer-dimer (or higher order oligomeric) equilibria, we labeled purified SLC17A9 with Alexa-Fluor 647 using N-hydroxyl succinimide (NHS) esterification and then mixed it with increasing amounts of unlabeled protein, followed by Microscale Thermophoresis, a method allowing for determining binding affinities between labeled and unlabeled specimens in solution under equilibrium conditions (28). Titrations resulted in migration differences that were used to generate a binding curve, yielding an apparent K_D_ of 12 μM for the monomer-dimer equilibrium (Fig. 2*B*). Note that the value is only a rough estimate since NHS-labeling is expected to result in a mixture of differently labeled protein species with randomly derivatized endogenous amino groups, and the presence of some unlabeled protein cannot be excluded.

In conclusion, our experiments confirm that SLC17A9 can be expressed in insect cells using the baculovirus system with various tags. Purification results in a full-length protein that is soluble in different detergents, stable and apparently folded, and that appears to reversibly form dimers or even higher order oligomers, although further confirmation is needed, particularly when considering that other members of the SLC17 family were reported to be monomeric in intact membranes (29). Since no differences were detectable between proteins containing either the N-terminal SBP-tag or the C-terminal Twin-Strep tag, both proteins were used interchangeably for further characterization.

### Characterization of ATP-uptake by SLC17A9 after reconstitution in artificial vesicles

To investigate whether SLC17A9 transports ATP in dependence of an electrochemical proton gradient, we reconstituted purified SLC17A9 protein together with a bacterial F_o_F_1_-ATPase (from *Bacillus thermophilus*, henceforth designed as TF_o_F_1_) in liposomes, following largely a procedure published previously for reconstituting the vesicular glutamate transporter VGLUT1 (30). The resulting liposomes were heterogeneous, with an average diameter of approximately 140 nm as determined by dynamic light scattering and by negative staining electron microscopy (Fig. S2*B*). We then immobilized a sample of the liposomes on glass slides, immunostained them for both SLC17A9 and TF_o_F_1_ and analyzed the degree of colocalization using STED-microscopy. More than 80% of all labeled particles contained both labels (Fig. S2*C*), showing that the vast majority of the liposomes were successfully co-reconstituted with both proteins.

We also checked the orientation of SLC17A9 after reconstitution. While no structure is available for SLC17A9, we assume, given the sequence similarity to VGLUTs, that both the C- and the N-terminal ends face the cytoplasm, a notion that is also in agreement with the structural prediction by AlphaFold 3 (Fig. S3*B*). To test whether this is also the case after reconstitution, we reconstituted SLC17A9 containing the SBP-tag in liposomes and then treated them with TEV protease either in the absence (intact liposomes) or presence of the detergent Triton X-100 (to expose all tags). As shown in Fig. S3, the vast majority of the SBP tag was cleaved off by TEV treatment of intact liposomes, showing that, similar to the related VGLUTs, the protein is mostly oriented with the N-terminus facing the outside.

Next, we investigated whether liposomes reconstituted with SLC17A9 can establish a stable protonmotive force ΔμH^+^ when TF_o_F_1_ is activated by adding ATP. In the absence of any compensatory ion movements, proton-pumping by the ATPase will generate an inside positive membrane potential which then stops further pumping of protons, preventing luminal acidification. However, if charge compensation across the membrane is permitted, *e.g.* by the efflux of cations (here K^+^), the membrane potential is diminished, allowing for the ATPase to resume proton pumping, which results in the establishment of a pH-gradient ΔpH. As shown in Figure 3, the TF_o_F_1_-containing liposomes established the expected gradients regardless of whether SLC17A9 was co-reconstituted or not: When adding ATP, an inside positive membrane potential was generated (measured with the reporter dye oxonol VI) that was dissipated by the protonophore FCCP (Fig. 3*A*). In contrast, no pH-change was detectable (measured with the reporter dye acridine orange). When the K^+^-ionophore valinomycin was added, compensatory K^+^-efflux occurs during proton pumping, resulting in the establishment of a ΔpH that was again dissipated by FCCP (Fig. 3*B*).

Stable pH gradients can also be generated by coupling them to diffusion potentials in the absence of the ATPase. To this end, the liposomes were loaded with 150 mM K^+^ and incubated in K^+^-free buffer, followed by addition of FCCP and valinomycin to permeabilize the membrane for both K^+^ and H^+^. Under these conditions, the outward-directed K^+^-gradient causes an inside-negative Δψ that is compensated by proton influx, resulting in vesicle acidification (Fig. 3*C*). Note that we also generated inside-positive Δψ by reconstituting liposomes in K^+^-free buffer, followed by incubation in a buffer containing 150 mM K^+^. Upon addition of valinomycin, potentials were generated not shown), but they were transient with a relatively short lifetime (not shown, see *e.g.* (31)).

We then tested whether the SLC17A9-containing liposomes were capable of transporting ATP. However, while an increase of ATP binding was observed at higher concentrations (that could be partially competed for by excess of unlabeled ADP), we were unable to observe any ΔμH^+^-dependent ATP-uptake, regardless of whether the potential was shifted predominantly towards Δψ (which was previously reported to drive transport as discussed above) or towards ΔpH (Fig. 4). Please also note that the protein was not stabilized by the presence of ATP (up to 10 mM) in thermal denaturation experiments (not shown), which is commonly observed upon ligand binding to a specific binding site.

Numerous control experiments were carried out to ensure that our assays were working properly. For instance, when reconstituting the related transporter VGLUT1 under comparable conditions, ΔμH^+^-dependent glutamate uptake, but no ATP uptake, was observed (Fig. S4). To exclude substrate competition, we measured ATP consumption by the TF_o_F_1_-ATPase at the end of the uptake experiments and found it to be negligible, *i.e.* not significantly different from the starting concentration (not shown). We also varied the molar lipid: protein ratio between 1000 : 1 and 4000 : 1, also to reduce carry-over of the detergent DDM that, at higher concentrations, resulted in some leakage of ATP (not shown). To further exclude detergent-based artefacts, we used glycoside detergents with shorter chain length that are easier to remove, including decyl maltoside (DM) and octyl glucoside (OG). Whereas purification and reconstitution in DM yielded results comparable to those of DDM, showing no ATP uptake despite stable membrane potentials, OG resulted in an at least partial inactivation of the TF_o_F_1_-ATPase and reduced solubility of the SLC17A9 protein. No evidence for gradient-dependent uptake was observed under any of these conditions. It also did not matter whether we used the protein variant containing the N-terminal SBP-tag or the C-terminal Twin-Strep tag. As an additional positive control, we prepared chromaffin granule ghosts (lysed chromaffin granules allowed to reseal). These vesicles showed FCCP-sensitive ATP uptake (Fig. S4), in agreement with previous results, confirming that our uptake assay was working (note that the SLC17A9 protein was not enriched in this fraction, in stark contrast to the V-ATPase and other chromaffin granule marker proteins).

Note that we also made major efforts to develop a fluorescent-based uptake assay that is more suitable for measuring uptake reliably at high ATP concentrations. To this end, we developed a protocol in which a purified ATP-sensor termed ATeam (32), carrying a His_14_ tag, was added to the reconstitution step. When the membrane lipids were supplemented with 2% of a nickel-containing membrane lipid (DGS-NTA(Ni), see Methods for details), efficient binding of the sensor to both the inside and outside of the liposome membrane was observed. The external pool was removed by treating the liposomes with EDTA and imidazol followed by density gradient centrifugation. The resulting liposomes were capable of generating pH-gradients and membrane potentials, *i.e.* essentially indistinguishable from the liposomes prepared by the standard protocol (see examples in Fig. 3). Control experiments revealed that ATP is only detected when ATP has access to the interior of the liposome or when the liposomes are disrupted by detergent. These data are not shown since also with this procedure we were unable to obtain any evidence for SLC17A9-dependent ATP transport, in agreement with the data described above.

### Does SLC17A9 function as a transporter for inorganic phosphate (P_i_)?

In addition to vesicular transporters of organic anions, the SLC17 family contains several Na^+^-dependent P_i_-transporters. Indeed, the vesicular glutamate transporter VGLUT1 was originally identified as a P_i_ transporter (33). It was shown later that VGLUT1 (and also the related VGLUT2 and 3) performs both functions in dependence of its localization and orientation (34, 35). In synaptic vesicles, with the C- and N-termini exposed to the cytoplasm, it functions as a ΔμH^+^-dependent glutamate transporter that is essential for glutamatergic neurotransmission. P_i_ is also transported in this mode and competes with glutamate for the same binding site, albeit with lower affinity. When inserted in the plasma membrane after exocytosis, it exposes its luminal domain to the extracellular surface and transports P_i_-ions into the cell, *i.e.* in the opposite direction of glutamate transport, in a manner that is strictly coupled to the cotransport of Na^+^-ions, with a stoichiometry of at least 2Na^+^: 1 P_i_ (35, 36). For these reasons, we tried to determine whether SLC17A9 functions as a P_i_ transporter in the inverted orientation.

To this end, we carried out two types of experiments. First, we transiently expressed SLC17A9 in HeLa cells together with eGFP (using a bicistronic IRES vector, controls eGFP only), resulting in robust expression of SLC17A9 (Fig. 5, *A* and *B*). Immunostaining revealed that the transporter is predominantly localized to intracellular vesicles, with minor pools in the plasma membrane (Fig. 5*B*). To determine whether the protein is involved in P_i_-transport, the cells were weaned from P_i_ by incubation in P_i_-free buffer for 1 h and then incubated in the presence of P_i_ for 15 min. Cells were then collected, washed, and the P_i_-content was determined using a colorimetric assay (see Methods). As shown in Figure 5*C*, no significant differences in Pi-content was observable between SLC17A9-expressing cells and cells transfected only with eGFP, although a slight but consistent trend towards sensitivity to clodronate treatment was observed in SLC17A9-expressing cells.

**Figure 6:**
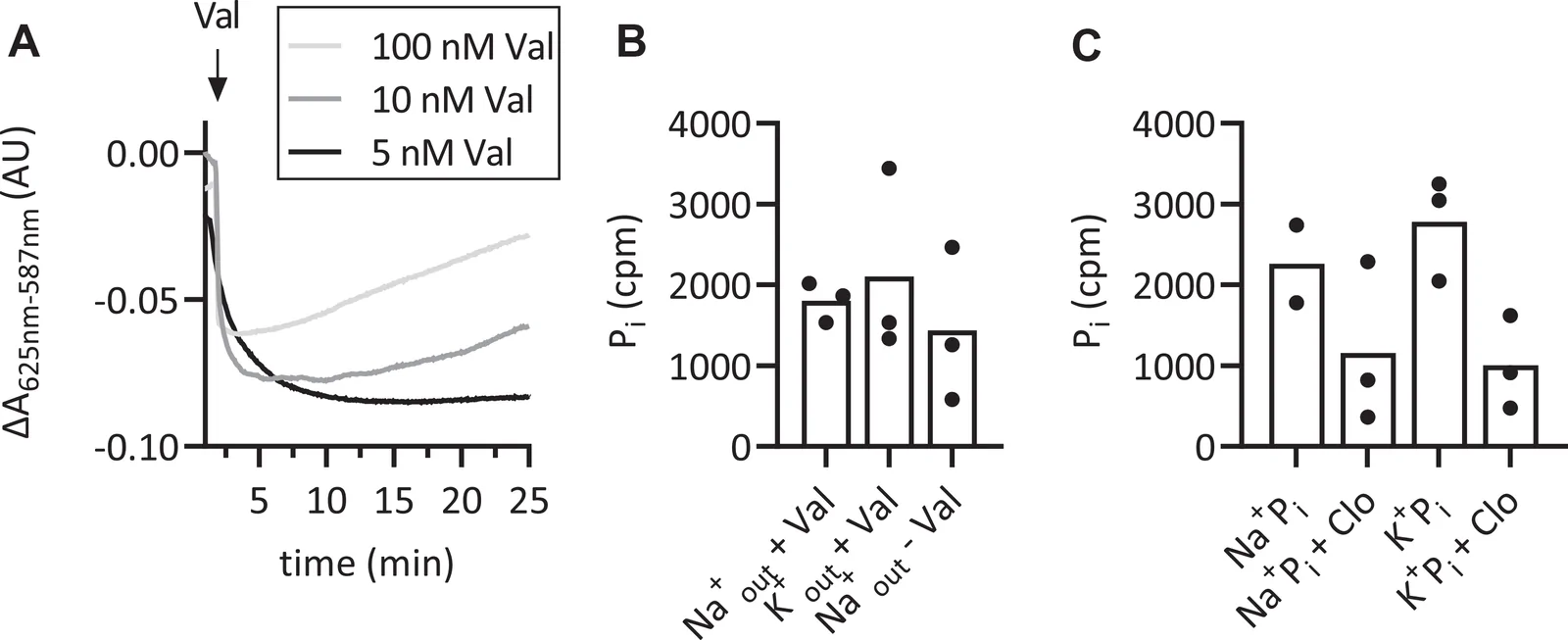
**Liposomes reconstituted with SLC17A9 protein bind P_i_ but do not show Na^+^ -dependent transport.***A*, generation of an inside negative diffusion potential by liposomes reconstituted with SLC17A9. The liposomes were prepared in 150 mM K^+^-gluconate buffer. The membrane potential was measured with Oxonol VI as in Figure 3. A strong potential change was observed upon the addition of 5 nM valinomycin, which was rendered unstable when the valinomycin concentration was increased. *B and C*, uptake of radiolabeled P_i_ by liposomes prepared as in (*A*). The reaction was started by the addition of valinomycin (Val), radiolabeled P_i_, and, where indicated, clodronate (Clo), and stopped after 2 min at 32 °C. For measuring Na^+^-dependent transport, K^+^-gluconate was exchanged for Na^+^-gluconate immediately before the assay. See methods for details. Data points of three independent experiments are shown.

In the second set of experiments, we reconstituted SLC17A9 in liposomes preloaded with K^+^ and then incubated them in buffers that were either containing Na^+^ or K^+^ ions, followed by adding the K^+^-ionophore valinomycin. When Na^+^ is present on the outside, valinomycin creates an inside negative membrane potential that provides a driving force for P_i_-uptake (Fig. 6*A*, note that if two or more Na^+^ ions are transported together with one P_i_-ion, the net charge of the transported ions is positive). However, unlike in our corresponding experiments on VGLUT, no Na^+^-dependent P_i_-transport was detectable (Fig. 6*B*). In contrast, addition of clodronate reduced P_i_-retention regardless of the presence of cations or valinomycin (Fig. 6*C*), suggesting that under these conditions the protein binds but does not transport P_i_-ions.

## Discussion

In the present study, we have revisited the proposed function of SLC17A9 as a secondary active transporter of ATP and related nucleotides and nucleosides using biochemical approaches. Despite major efforts and many control experiments, we were unable to reproduce the transport activity published previously by Moriyama and colleagues as outlined in the Introduction.

At this point, we have no convincing explanation for this discrepancy. The evidence supporting the view that SLC17A9 is indeed identical with the long sought-after nucleotide transporter of secretory vesicles and other ATP-containing intracellular compartments is quite strong (for review see (25)): It includes both *in vitro* reconstitution of active transport from purified components as well as knockout and knockdown studies, some of which were carried out independently by different laboratories and show a reduction in ATP release from various cell types. Also, despite the many control experiments discussed above, it can never be excluded in such biochemical experiments that “something went wrong experimentally”, *that is*, that one critical parameter that remained unnoticed in protein expression and purification, liposome reconstitution, or transport assays, was responsible for the loss of activity in our hands, which should have been preventable when appropriate procedures were followed.

With these caveats in mind, we searched for experimental details that differ between our and the previously published work. First, we noted that the apparent molecular mass, as determined by SDS-PAGE, of the protein purified here is lower than that reported previously. N-Glycosylation is unlikely to be the cause since the only consensus site is inside a predicted transmembrane domain. In addition, in more recent studies by Moriyama and coworkers, the protein was expressed in bacteria rather than in eukaryotic cells, yielding again apparent Mr values between 60 and 70 kDa. However, membrane proteins may not completely unfold in SDS unless heated to 100 °C, leading to different migration behavior upon SDS-PAGE. Indeed, our analysis by blue-native electrophoresis reveals that detergents have a major influence on the migration behavior in polyacrylamide gel electrophoresis. Moreover, it is conceivable that under certain conditions a dimer is stabilized in SDS-PAGE, which we also observed occasionally (see *e.g.* Fig. S3). As outlined above, most of our experiments were performed with SLC17A9 purified in DDM instead of octylglucoside since, in our hands, the protein was less stable and tended to precipitate in OG. Second, in our study, the concentrations of valinomycin for generating diffusion potentials were at least one order of magnitude lower (see also (37)) than those published by (11), as in our hands the potentials were unstable and quickly dissipated at higher valinomycin concentrations. Also, the lipid mixes used for reconstitution were different (we used a mixture of purified lipids corresponding to brain lipids instead of soybean lipids, because in previous experiments we noted lower activity of VGluTs in soybean lipids). Moreover, we use filtration instead of size-exclusion spin columns for separating free ATP from vesicles, which we found to be more reliable based on our previous experience with reconstituting VGLUTs. Although we consider it as unlikely, we cannot rule out that one of these factors is responsible for, or contributed to, the discrepant observations.

We were also unable to clarify whether SLC17A9 functions as a P_i_-transporter when localized at the plasma membrane, thus resembling most of the other SLC17A transporters. Again, and in contrast to our previous work on VGLuT1, we did not observe Na^+^-dependent transport in liposomes, but this may be explained by the fact that the percentage of liposomes containing the transporter in the inverse orientation (as required for Na^+^-dependent P_i_-uptake in liposomes, see (35) was too low to yield detectable signals. Also, we cannot exclude that upon expression of SLC17A9 in HeLa cells the amount of transporter localized in the plasma membrane was too low for resulting in detectable changes in phosphate accumulation. In this context it is interesting to note that SLC17A9 appears to be localized on intracellular vesicles enriched in pyrophosphate and higher-order polyphosphates (38), but it needs to be clarified to which extent the transporter is involved in their uptake or storage.

We conclude that there are open questions concerning the transport function of SLC17A9 that warrant further investigation. Considering the strong evidence supporting a key role of SLC17A9 in vesicular ATP storage, we cannot exclude that a critical component is missing, such as an additional protein, that is required for active transport of ATP into vesicles. On the other hand, the fact that knockout of SLC17A9 results in only very mild phenotypes is difficult to reconcile with the central role of vesicular ATP as a standalone neurotransmitter or its crucial role in the storage of other transmitters unless there are other ways to compensate for its deficiency. Finally, considering the ubiquitous distribution of the protein in virtually all tissues, it cannot be excluded that the protein functions in a broader context, perhaps transporting other metabolites, or else is functionally redundant to other transporters that yet need to be identified.

## Experimental procedures

### Subcellular fractionation

Synaptic vesicles (SVs) were isolated from rat brain as described previously, using both a vesicle-enriched fraction obtained by differential centrifugation after hypotonic lysis of synaptosomes (fraction LP2), and a highly purified fraction obtained after a final size exclusion chromatography on controlled-pore glass beads (fraction CPG-SVs) (39, 40). Ghosts of chromaffin granules were prepared according to standard procedures (41, 42). Final fractions were snap-frozen in liquid nitrogen and stored at −80 °C before use. Rats were kept in the Animal Care Facility of the Max-Planck Institute for Multidisciplinary Sciences, with all procedures in compliance with the German Animal Welfare Act (Tierenschutzgesetz, TierSchG) and the Animal Welfare Laboratory Animal Regulations (TierSchVersV).

### Expression and purification of recombinant proteins

The ATP-synthase from *B. thermophilus* (TF_o_F_1_-ATPase) was expressed in *E. coli* and purified as described previously (43) and stored in 10% glycerol at −80 °C until use.

SLC17A9 protein was expressed in Sf9 insect cells using the baculovirus expression system. Sf9 cells were transfected with an SBP-tagged SLC17A9 construct using pFastBac1 plasmid to produce virus that is then used to infect High5 insect cells in a suspension culture and monitored for viability every 24 h. The cells were harvested when the viability reached between 85% and 90%. The entire procedure was carried out as described in the instructions of the manufacturer (Thermo Fisher Scientific) following previously published procedures (44).

Harvested cells were suspended in 20 mM Hepes, pH 7.5, 150 mM NaCl, 10 mM MgCl_2_, 0.1 mM TCEP, DNAse and an EDTA-free protease inhibitor cocktail (cOmplete, Sigma-Aldrich). For each preparation roughly 20 gm of cells were resuspended in 100 ml of resuspension buffer at 4 °C and disrupted by passing three times through a microfluidizer at 15KPsi. Broken cells were centrifuged at 2000*g* for 10 min at 4 °C. The supernatant was collected and centrifuged 1 h at 190,000 g_max_ to pellet membrane vesicles. Membrane vesicles corresponding to 1 g of protein were resuspended in 20 ml solubilization buffer (20 mM Hepes, pH 7.5, 150 mM NaCl, 10 mM MgCl_2_, 0.1 mM TCEP, protease inhibitor cocktail, 20% glycerol, 2% DDM and 0.2% cholesterylhemisuccinate) and incubated under rotation for 1 h at 4 °C. The suspension was centrifuged at 250,000 g_max_ for 30 min. The supernatant was collected and mixed with 1 ml of streptavidin beads (obtained from Thermo-Fisher) that were washed with the solubilization buffer. After incubation for 3 h at 4 °C under rotation, the beads were collected in a small column and washed by gravity flow with 20 ml of wash buffer (20 mM Hepes, pH 7.5, 300 mM NaCl, 0.1 mM TCEP, 20% sucrose, 0.02% DDM). The SLC17A9 protein was eluted from the beads using 5 ml of elution buffer (20 mM Hepes, pH 7.5, 150 mM NaCl, 0.1 mM TCEP, 20% sucrose 0.02% DDM and 20 mM biotin).

The purification of twin-strep–tagged SLC17A9 was carried out following the protocol used for SBP-tagged protein, with minor modifications. Membrane vesicle solubilization was performed as described above. After centrifugation, the supernatant was collected and incubated with 0.5 ml of Strep-Tactin beads (Thermo Fisher, washed with solubilization buffer) for 3 h at 4 °C under gentle rotation. The beads were then transferred to a small column and washed with 10 ml of wash buffer (20 mM HEPES, pH 7.5, 300 mM NaCl, 0.1 mM TCEP, 20% sucrose, 0.02% DDM). The SLC17A9 protein was eluted from the beads using 2.5 ml of elution buffer (20 mM HEPES, pH 7.5, 150 mM NaCl, 0.1 mM TCEP, 20% sucrose, 0.02% DDM, and 50 mM biotin). Mass-spectrometric sequencing of purified SLC17A9 was carried out as described (45) after running the purified protein on an SDS-polyacrylamide gel and excising the corresponding protein band.

Size exclusion chromatography (SEC) was performed at 4 °C using a Superose 6 Increase 3.2/300 pre-packed column (2.4 ml bed volume; ∼0.8 ml void volume) on an ÄKTA go system (Cytiva). The column was equilibrated with SEC buffer (20 mM HEPES pH 7.5, 150 mM NaCl, 0.1 mM TCEP, and 0.02% DDM). Purified protein (100 μg at 1 mg/ml) was loaded onto the column and eluted at a constant flow rate of 50 μl/min. Elution was monitored by UV absorbance at 280 nm.

The protein eluted at an elution volume of 1.3 ml. The elution profile showed a dominant single peak, indicating a high degree of monodispersity and the absence of high-molecular-weight aggregates at the void volume. There is also a shoulder peak visible, which might represent the dimer fraction of the protein. Peak fractions between 1.25 and 1.35 ml elution volume were pooled and concentrated (1 mg/ml) and stored for further analysis.

VGLUT1 was expressed and purified as described previously (43).

### MicroScale thermophoresis and thermal denaturation

To determine the oligomeric state of the protein, MST experiments were performed using a Monolith NT.015 system (NanoTemper Technologies). . This method utilizes thermophoresis, the movement of molecules driven by a temperature gradient. By creating a local temperature difference (ΔT), the system induces a change in molecular concentration—either depletion or enrichment—which is measured by the Soret coefficient (ST) using the formula: chot/ccold = exp(-STΔT) (46, 47). In this experiment labeled SLC17A9 protein was titrated with unlabeled SLC17A9 protein. For labeling, purified SLC17A9 was derivatized with NHS-Alexa-Fluor 647 following the protocol of the manufacturer (Thermo Fisher Scientific) (48). During the measurement, the concentration of RED-NHS labelled SLC17A9 was kept constant at 1 to 2 nM concentration while the concentration of the ligand, which in this case was unlabeled SLC17A9 protein, was varied using consecutive two-fold dilution steps, with 38 μM as highest and 2.32 nm (16-fold dilution) as lowest concentration in elution buffer except biotin. Note that the concentration of labeled protein can vary dependent upon the extent of labeling and concentration of the ligand can vary depending upon binding affinities (K_D_ values). 10 μl sample for each titration was loaded in premium capillaries provided by NanoTemper Technologies, with overall 16 of such capillaries being loaded in a sample tray. A focused IR laser is used to heat the sample. Thermophoresis of the fluorescent SLC17A9 is measured along the temperature gradient. For measurement, the excitation power (laser intensity) and MST power was adjusted to 20% and 90% respectively.

For determining heat stability (melting), purified SLC17A9 was subjected to thermal denaturation. Changes in intrinsic fluorescence were recorded using a Prometheus instrument (NanoTemper Technologies), with 10 μl of protein sample being loaded in a capillary. As the protein unfolds due to increasing temperature, changes in the local environment of tryptophan cause shifts in their fluorescence properties which is measured by the ratio of fluorescence intensities (350 nm/330 nm, excitation at 280 nm). SLC17A9 protein was purified in different detergents including DDM, LMNG and GDN, and the corresponding melting temperatures were measured. The temperature increased from 30 °C to 90 °C, with the scan rate being set to 1 °C/min. Protein concentrations were varied for different samples.

### Preparation of proteoliposomes

Proteoliposomes were prepared largely following our previous protocol (30), with some adjustments. The following membrane lipids were mixed from stock solutions solubilized in chloroform: 1,2-dioleoyl-*sn*-glycero-3-phosphocholine (DOPC, 65 mol %), 1,2-dioleoyl-*sn*-glycero-3-phosphoserine (DOPS, 10 mol %) and cholesterol (25 mol %), all obtained from Avanti Polar Lipids (Alabaster, AL). In some experiments (Fig. 3, *B* and *C*) the lipid mix was supplemented with 2% 1,2-dioleoyl-sn-glycero-3-[(N-(5-amino-1-carboxypentyl)iminodiacetic acid)succinyl] (nickel salt) (DGS-NTA(Ni), also obtained from Avanti, with no discernible effects on the properties of the liposomes. The lipids were dried under a stream of nitrogen and further exposed to vacuum for at least 10 h to remove traces of chloroform. Unless indicated otherwise, the dried lipid mixture was resuspended in K^+^ gluconate (150 mM), MgCl_2_ (2 mM), and MOPS (10 mM), adjusted to pH 7.3 (K^+^gluc buffer) to yield a final lipid concentration of 16 mM. The multilamellar liposomal suspension was manually extruded 33 times with the Avanti Mini-Extruder (Avanti Polar Lipids) through a 100 nm polycarbonate track-etched membrane (Sigma-Aldrich) to yield unilamellar liposomes that were adjusted to a lipid concentration of 4 mM with K^+^gluc buffer.

For reconstitution of proteins, the liposomes were destabilized by gradually adding *n*-octyl-β-d-glucopyranoside (OG) under continuous mixing until a final concentration of 25 mM was reached. Where indicated, TF_o_F_1_ and the indicated vesicular transporter were added to the mixture to reach final concentrations of 0.1 μM (TF_o_F_1_), 2 μM (VGLUT and the TwinStrep tagged SLC17A9), and 1 μM (SBP-tagged SLC17A9), respectively, and incubated for 1 h at 25 °C and 650 rpm, followed by dialysis with a Slide-A-Lyzer 10,000 MWCO dialysis cassette (Thermo Fisher Scientific) against K^+^gluc buffer at 4 °C, initially for 4 h and after buffer exchange overnight, with 2 g BioBeads SM-2 (Bio-Rad Laboratories) added per liter of dialysis buffer. If an exchange of the external buffer of the proteoliposomes was required, PD-10 Sephadex G-25 Desalting columns (GE Healthcare Life Sciences) were used according to the manufacturer’s instructions.

In some of the experiments, a His_14_-tagged version of the fluorescent ATP-sensor ATeam (32) was encapsulated in the proteoliposomes. To this end, 2% of DGS-NTA(Ni) lipids (Avanti Polar Lipids) were included in the lipid mixture. During reconstitution 2 μm of His_14_ATeam were added together with the TF_o_F_1_ and the vesicular transporter, resulting in binding of the sensor to the DGS-NTA(Ni) lipids both inside and outside of the liposome membrane. The proteoliposomes were treated with 400 mM imidazole (pH 7.4) and 20 mM EDTA (pH 8,0) for 30 min at 4 °C to remove the ATP sensor facing towards the outside of the proteoliposomes. The proteoliposomes were separated from the free His_14_ATeam, TF_o_F_1_ and vesicular transporter by flotation on a Nycodenz density gradient. Nycodenz was removed by dialysing the collected proteoliposomes against K^+^ gluc buffer for 1 h at 4 °C with a Slide-A-Lyzer 10,000 MWCO dialysis cassette (Thermo Fisher Scientific).

### Characterization of proteoliposomes

Dynamic light scattering (DLS) was used to analyse the size distribution of the reconstituted proteoliposomes (49). The DLS measurements (diluted to be in the linear range of the instrument of 10^5^ -10^6^ counts) were carried out with the DynaPro Dynamic Light Scattering Instrument (Wyatt Technology) at 25 °C and a laser power of 5% in a 1.5 mm cuvette (Wyatt Technology). Negative staining electron microscopy was performed as described previously (50).

To assess to which extent the TF_o_F_1_ and SLC17A9 proteins overlap in the same liposome population, immunolabeling of the proteoliposomes was carried out as described ((51) followed by stimulated emission depletion (STED) microscopy. Briefly, proteoliposomes were immobilized on a coverslip and fixed with 4% paraformaldehyde (PFA). The proteoliposomes were permeabilized with 0.3% Triton X-100 for 5 min and immunolabeled against TF_o_F_1_ and SLC17A9. Imaging was performed using a STED microscope (Abberior Instruments). Particles with a size of 60 to 500 nm were analyzed for TF_o_F_1_ and SLC17A9 occurrence using Fiji.

To test for the orientation of SLC17A9 after reconstitution, proteoliposomes were treated with 3 μM TEV protease C (kindly provided by Dr Alexander Stein’s laboratory, MPI-NAT, Göttingen) for 1 h at 25 °C and under continuous rotation in order to cleave off the N-terminal SBP tags exposed to the outer surface of the liposomes. Control incubations included solubilization of the liposomes in 0.1% TritonX-100 prior to proteolysis, and incubations from which TEV protease C was omitted. Samples were analyzed by SDS-PAGE followed by immunoblotting for the SBP-tag.

Acidification measurements and measurements of changes in the membrane potential were carried out using the reporter dyes acridine orange and oxonol VI, respectively (both from Molecular Probes) as described previously (31, 52).

### Measurement of radiolabeled ATP, glutamate, and P_i_ uptake

The assays for the uptake of radiolabeled substrates were carried out mostly as described previously: glutamate (52, 53), ATP (16), and P_i_ (35), with some adjustments.

[^3^H]glutamic acid (specific activity: 60 Ci/mmol, concentration: 1 mCi/ml), [^3^H]ATP (specific activity: 25 Ci/mmol, concentration: 1 mCi/ml) and [^33^P]phosphoric acid (specific activity: 3000 Ci/mmol, concentration: 5 mCi/ml) were purchased from Hartmann Analytic (Braunschweig, Germany). Glutamate uptake by proteoliposomes and SVs was measured using 6 μCi [^3^H]glutamic acid per sample in the presence of 4 mM Mg^2+^ATP (pH 7.3), 4 mM KCl and 100 μM K^+^glutamate (uptake buffer). ATP uptake was measured using 5 μCi [^3^H]ATP per sample in uptake buffer, with the Mg^2+^ATP (pH 7.3) and KCl concentrations adjusted as indicated. P_i_ uptake was measured using 2 μCi [^33^P]phosphoric acid per sample in the presence of 0.6 mM Mg^2+^ATP (pH 7.3), and KCl and unlabeled P_i_ as indicated.

The uptake reaction was initiated by mixing 90 μl of sample with 10 μl of uptake buffer (10×, *i.e.*. containing all reagents at 10x concentration), followed by 15 min incubation at 32 °C. The protein amounts used per assay included 15 μg for LP2, 10 μg for CPG-SVs or 100 μg for CGGs. For proteoliposomes, amounts corresponding to 9 μg protein of either VGLUT1 or SLC17A9 were added.

The reaction was stopped by pipetting the reaction mixture into 3 ml of ice-cold K^+^gluc buffer. Proteoliposomes, SVs and CGGs containing radiolabeled substrate were then rapidly captured by vacuum filtration on a 0.45 μm mixed cellulose ester membrane filter (Merck Millipore, Billerica, MA). The filter was washed three times with 3 ml ice-cold buffer and submerged in the Ultima Gold liquid scintillation cocktail (PerkinElmer, Waltham, MA). The radioactivity was quantified with the liquid scintillation analyzer Tri-Carb 2100TR (Canberra Packard, Schwadorf, Austria). Note that for measuring [^3^H]ATP uptake, the membrane filters were soaked in 1% bovine serum albumin (BSA) and washed once with buffer before the samples were applied to reduce non-specific binding of the free ligand. When [^3^H]ATP or [^3^H]glutamic acid were used, membranes were incubated in the scintillation cocktail overnight prior to counting for radioactivity to maximize ^3^H-recovery.

For measuring P_i_-transport, the liposomes were reconstituted in 150 mM K^+^gluconate, 10 mM MOPS, pH 7.3. To check for Na^+^-dependence of P_i_-transport, K^+^-gluconate was exchanged with 150 mM Na^+^-gluconate directly before the assay. The assay was initiated by adding 5 nM valinomycin, 0.5 mM Na^33^P_i_ (2 μCi/data point) and incubated for 2 min at 32 °C. Where indicated, 100 μM clodronate was included.

### P_i_ uptake measurements in SLC17A9-expressing HeLa cells

HeLa cells were grown in Dulbecco’s Modified Eagle’s Medium (DMEM, Sigma-Aldrich) with 4.5 g/l glucose, 10% fetal bovine serum (PAA Laboratories), 2 mM glutamine, 100 U/ml penicillin and 100 μg/ml streptomycin (Gibco Life Technologies) at 37 °C and 5% CO_2_.

HeLa cells were transfected with the plasmid pIRES2-EGFP carrying the DNA sequence of SLC17A9 (*Mus musculus*). As a negative control, HeLa cells were transfected with the empty pIRES2-EGFP, referred to as MOCK.

For immunostainings, HeLa cells were seeded onto 18 mm coverslips pre-coated with 0.1 mg/ml poly-D-lysine and grown at 37 °C and 5% CO_2_ to a confluency of 80%. Per coverslip, 1.5 μl of Lipofectamine 3000 (Invitrogen) in 250 μl serum-free DMEM and 1 μg of plasmid DNA in 250 μl serum-free DMEM with 2 μl P3000 (Invitrogen) were separately prepared and incubated for 5 min. Afterwards, the Lipofectamine 3000 and plasmid solution were combined and incubated for 20 min. The HeLa cells were transfected with 500 μl of the transfection mixture for 20 h at 37 °C and 5% CO_2_ before they were fixed, stained and imaged.

For the P_i_ uptake measurements and membrane fraction isolation, HeLa cells were seeded on 10 cm cell culture dishes and grown to a confluency of 80%. Each dish was transfected with 12 μl of Lipofectamine 3000, 7 μg plasmid DNA and 10 μl P3000 in 2 ml serum-free DMEM for 20 h, following the same procedure described above. P_i_ uptake was measured as described previously (36).

To prepare a membrane fraction of HeLa cells, the cells were first washed with PBS, collected by centrifugation in lysis buffer (150 mM NaCl, 50 mM Tris, pH 7.3) and frozen at −80 °C. After thawing and resuspension in lysis buffer supplemented with EDTA-free P_i_, the cells were disrupted with 12 cycles of 10 s sonication (50% duty cycle; Branson Sonifier 450, BRANSON Ultrasonics). The lysate was centrifuged for 5 min at 4 °C and 10,000 rpm to remove debris and large particles (Heraeus Biofuge Fresco, Thermo Fisher Scientific). The supernatant was subjected to ultracentrifugation for 20 min at 4 °C and 40,000 rpm (rotor: S45-A, Thermo Fisher Scientific), and the pelleted membranes were resuspended and analyzed by SDS-PAGE and immunoblotting.

### Fixation, immunostaining, and microscopy

Immunocytochemistry of HeLa cells largely followed standard procedures. The protocol described below is adapted from the webpage of Synaptic Systems (www.sysy.com). Briefly, cells were washed twice in phosphate-buffered saline (PBS), then fixed with PFA 4% for 10 min at RT. Samples were then washed three times in PBS containing 0.1% (v/v) Triton X-100 and then blocked in PBS/goat serum/Triton X-100 (89.9/10/0.1%) for 1 h at RT under gentle agitation. Incubation with primary antibodies was performed in PBS/goat serum/Triton X-100 (98.9/1/0.1%) overnight at 4 °C. Cells were then washed three times with PBS/Triton X-100 (99.9/0.1%), and incubated with the secondary antibodies for 1 h at RT under agitation. Cells were washed three times, then mounted in Fluorescence Mounting Medium (Dako Agilent Technologies) and stored at 4 °C. Image acquisition was performed using a Leica LSM700 confocal microscope. The following antibodies were used: anti-VGLUT1 (guinea pig polyclonal, Synaptic Systems, dilution 1:1000), anti-V-ATPase (polyclonal, Synaptic Systems), anti-SLC17A9 (VNUT) (guinea pig polyclonal, Merck Millipore, dilution 1:1000), and for detection a Cy3-labeled donkey-anti-guinea pig antibody obtained from Sigma-Aldrich (dilution 1:500).

### P_i_ uptake measurements in HeLa cells

Measurements of P_i_ uptake in HeLa cells were carried out essentially as described previously for PC12 cells (35, 36) except that the depolarization–repolarization steps were omitted.

HeLa cells transfected with SLC17A9-IRES-EGFP or MOCK were grown to near-confluency and starved for P_i_ by incubating for 1 h in P_i_-free KREBS buffer containing 4.7 mM [K^+^]. After two washing steps with P_i_-free buffer, the cells were detached *via* scraping, collected by centrifugation, and resuspended in 200 μl of P_i_-free KREBS buffer per 10 cm cell culture dish.

P_i_ uptake was initiated by incubating 50 μl of the cell suspension in KREBS buffer supplemented with 10 mM Na^+^ P_i_ (adjusted to a pH of 7.4) for 15 min at 37 °C and 700 rpm (ThermoMixer, Eppendorf SE). For SLC17A9-expressing cells, 100 μM clodronate (Merck) was used as an inhibitor. The reaction was stopped on ice, and the cells were centrifuged for 30 s at 4 °C and 12,000 rpm (Heraeus Biofuge Fresco, Thermo Fisher Scientific). The cells were washed three times with 200 μl ice-cold Pi-free KREBS buffer. To extract P_i_, cells were resuspended in 200 μl ddH_2_O followed by heating at 95 °C for 2 min under vigorous shaking. The lysates were centrifuged for 1 min at room temperature and 12,000 rpm (Heraeus Biofuge Fresco, Thermo Fisher Scientific). The P_i_ content of the supernatant was measured using the BIOMOL GREEN reagent (Enzo Life Sciences, Lausen, Switzerland) following the manufacturer’s instructions. The Tecan Genios Pro plate reader (Tecan Group AG) was used to measure the absorbance at 650 nm. The P_i_ uptake was normalized to the corresponding uptake conditions lacking Na^+^ P_i_.

### Other methods

Standard procedures include SDS-PAGE using Tricine gels (54), blue-native PAGE (27), and immunoblotting (55). Protein was determined using the PIERCE 660 nm protein-assay-kit (Thermo Fisher Scientific).

## Data availability

The original data are available on *Edmond* – the open research data repository of the Max Planck Society: https://edmond.mpg.de/.

## Supporting information

This article contains supporting information (42).

## Conflict of interest

The authors declare the following financial interests/personal relationships which may be considered as potential competing interests:

Some of the antibodies used in this study were obtained from the Company Synaptic Systems (Goettingen, Germany). Reinhard Jahn is one of three owners/shareholders of this company. Moreover, Marcelo Ganzella joined the company Synaptic Systems after completing a postdoctoral period in the laboratory of Reinhard Jahn.
