# Supplementary material for: Does SLC17A9 (VNUT) function as a stand-alone nucleotide and/or phosphate transporter?
Source: J Biol Chem. 2026 Jul 22;302(9):113360. doi: 10.1016/j.jbc.2026.113360 (PMC13521087; doi:10.1016/j.jbc.2026.113360)
Supplement: Supplementary file 1 [file mmc1.pdf]

Supplementary Material

Supplementary Fig. 1: Recombinant SLC17A9 protein is folded, with defined thermal unfolding transitions in different detergents

Determination of the melting temperature of SLC17A9 protein purified in different detergents as indicated in the figure. Purified SLC17A9 protein was subjected to thermal denaturation, and change in intrinsic fluorescence was recorded using Monolith NT.115 (NanoTemper Technologies GmbH, Munich, Germany) where 10 µL of protein was loaded in a capillary. As the protein unfolds due to increasing temperature, changes in the local environment of tryptophan cause shifts in their fluorescence properties which is measured by the ratio of fluorescence intensities (350 nm/330 nm). All samples contain 0.002 % CHS lipid, and protein concentrations were 2.5 mg/ml for DDM and LMNG samples, 4.0 mg/ml for amphipoles and 3.1 mg/ml for GDN samples. In each figure, the grey curve represents the ratio of fluorescence intensities (350 nm/330 nm) and the black curve represents its first derivative. The transition temperatures (peak value of the first derivative) are indicated, note that no defined transition temperature was obtained for the protein purified in amphipole A8-35.

Supplementary Fig. 2: Size distribution of proteoliposomes reconstituted with SLC17A9 and TF_oF_1

A: Left: Histogram of the size distribution determined by dynamic light scattering. Right: Representative electron micrograph of liposomes visualized by negative staining (scale bar: 200 nm). On the left, a magnified liposome (scale bar: 50 nm) contains a particle on the surface that probably represents the F_oF_1 ATPase (marked with a white arrow). Note that in this experiment, the liposomes were also loaded with a fluorescent ATP-sensor (see Methods).

B: Left: Liposomes, adsorbed on a slide, fixed and immunostained for SLC17A9 and TF_oF_1, imaged by STED microscopy. Right. Quantification of liposomes immunopositive for SLC17A9, TF_oF_1, and both, revealing that the majority of the liposomes contains both proteins. The experiment was repeated three times, and a total of 42 images were used for quantification. Each image was separately analyzed, and mean values and SEM were calculated (n=42).

Note that in this experiment, liposomes preloaded with ATeam were used.

Supplementary Fig. S3: The N-terminal domain of SLC17A9 is mostly exposed on the outer surface of the proteoliposomes

Liposomes reconstituted with SBP-tagged SLC17A9 were incubated as indicated for 1 h at 25°C in the presence and absence of TEV-protease, either intact or solubilized by Triton X-100. The samples were analyzed by immunoblotting and probed with an antibody specific for the SBP tag. TEV-cleavage abolished most of the signal in intact liposomes, with only a slight further enhancement being visible when Triton was added. Note that detergent treatment shifts the protein band more toward approx. 75 kDa suggesting that under these conditions the dimer is stabilized.

B: Structure of SLC17A9 (monomer), as predicted by AlphaFold 3. For clarity, the N-terminal region including the 6 N-terminal predicted transmembrane domains are coded in blue, whereas the C-terminal part is shown in black.

Supplementary Fig. S4: Uptake assays in liposomes and purified chromaffin granule ghosts show transport activity, confirming that the assays are working.

A, B: Liposomes reconstituted with SLC17A6 (VGLUT1) but not SLC17A9 accumulate glutamate when the TF_oF_1 ATPase is activated, showing that VGLUT remains functional under our purification and reconstitution conditions, and that SLC17A9 does not transport glutamate.

C: Chromaffin granule ghosts (CGG, 100 µg protein/data point), prepared after lysing and resealing of purified chromaffin granules (CG), show FCCP-sensitive uptake of ATP. In contrast, no activity was detectable in an enriched synaptic vesicle fraction (LP2, 15 µg/data point).

D: Immunoblot showing the distribution of the V-ATPase and SLC17A9 in fractions obtained by subcellular fractionation of homogenates of bovine adrenal medulla that were used to purify CGGs. Whereas the V-ATPase shows strong enrichment in the final fraction, SLC17A9 was detectable but not enriched. S1, supernatant obtained by clearing the homogenate of cell debris and nuclei (P1), P2, medium-speed pellet containing mostly chromaffin granules and mitochondria (S2 is the resulting supernatant), CG, fraction enriched in chromaffin granules after spinning P2 through a continuous sucrose density gradient, CGG, chromaffin granule ghosts obtained by centrifugation after osmotic lysis of CG, resulting in the loss of content proteins. See (Park et al., 2012) for details.

Park, Y., J.M. Hernandez, G. van den Bogaart, S. Ahmed, M. Holt, D. Riedel, and R. Jahn. 2012. Controlling synaptotagmin activity by electrostatic screening, *Nat Struct Mol Biol*., **19**, 991-997.

Figure S1

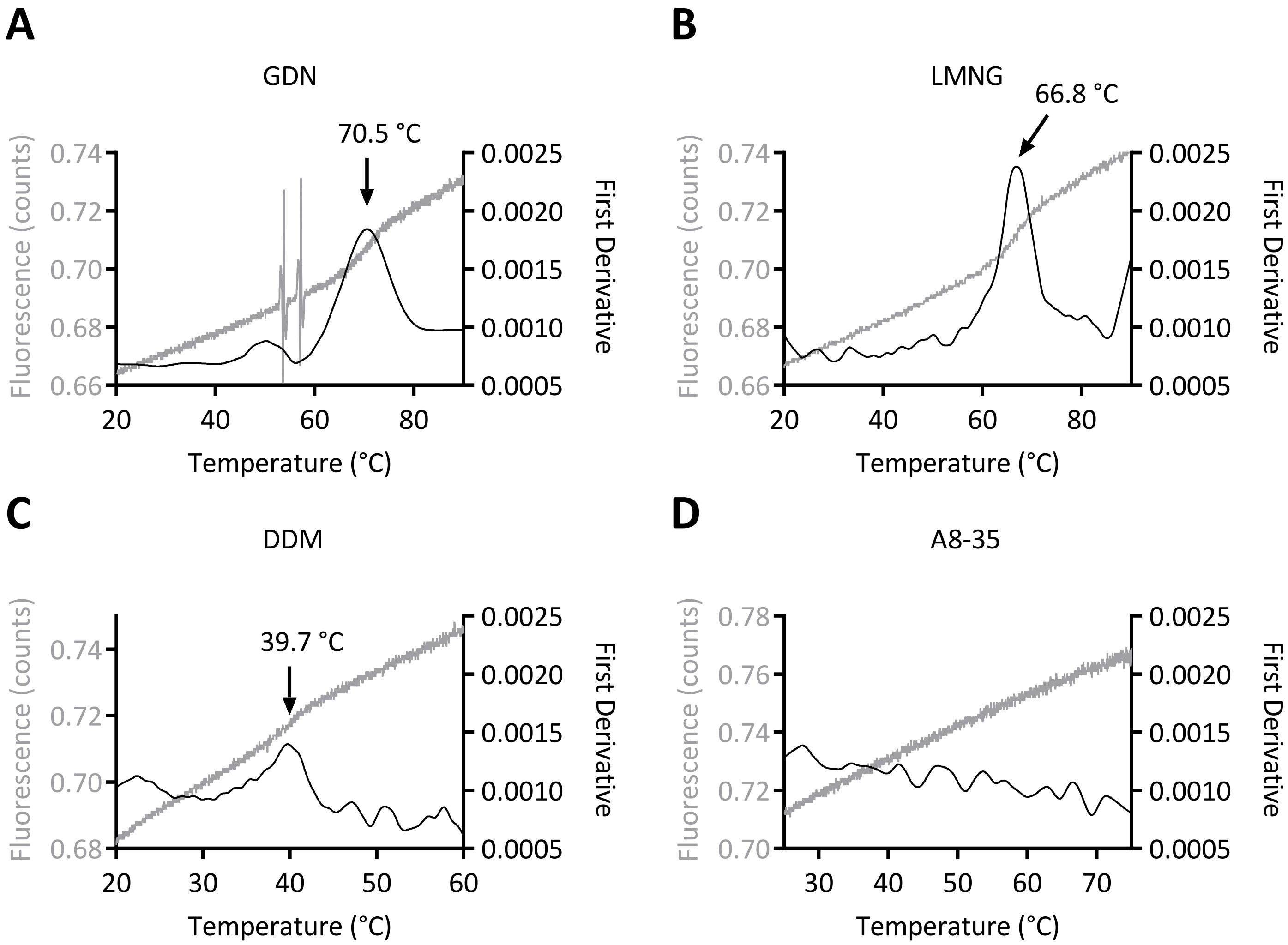

A
GDN
70.5 °C
Fluorescence (counts)
0.74
0.72
0.70
0.68
0.66
0.0025
0.0020
0.0015
0.0010
0.0005
First Derivative
20
40
60
80
Temperature (°C)
B
LMNG
66.8 °C
Fluorescence (counts)
First Derivative
Temperature (°C)
C
DDM
39.7 °C
Fluorescence (counts)
First Derivative
30
50
Temperature (°C)
D
A8-35
Fluorescence (counts)
0.78
0.76
First Derivative
70
Temperature (°C)

Figure S2

A

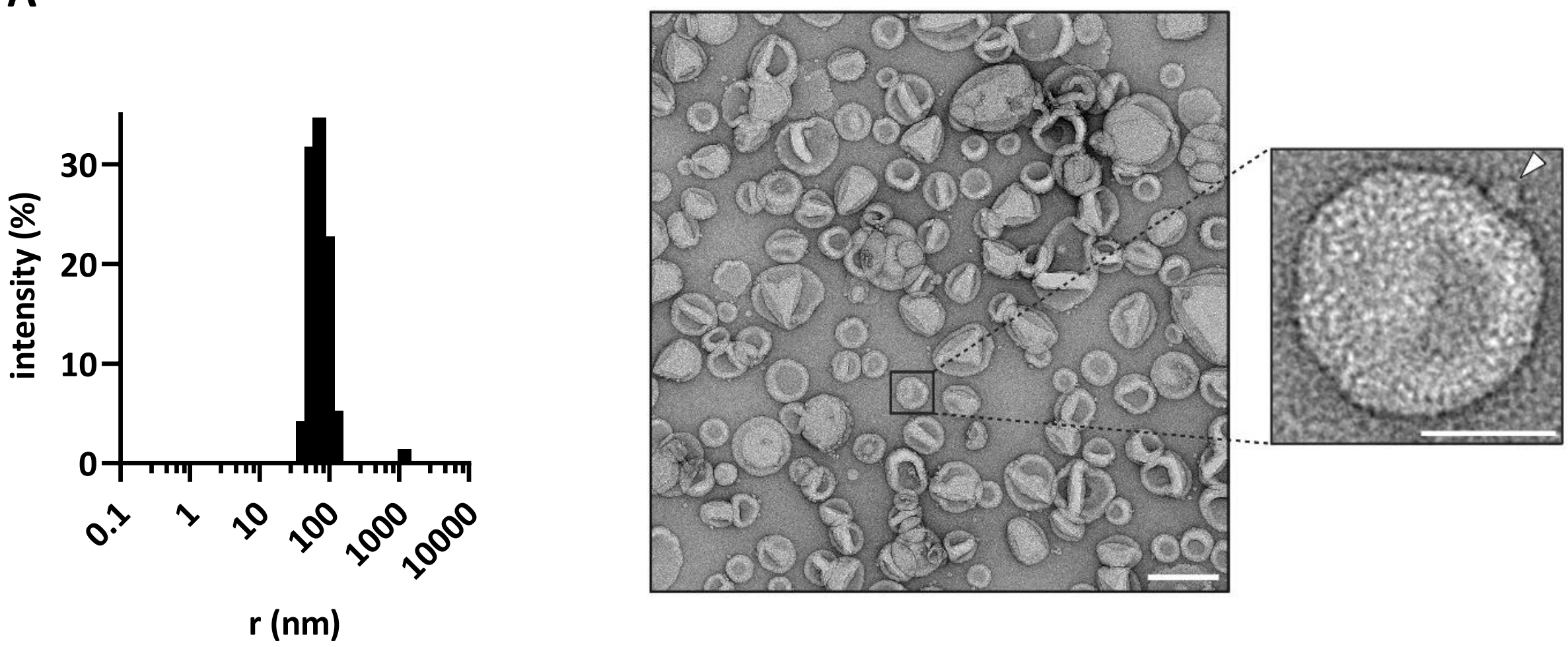

intensity (%)
30
20
10
0
0.1
1
10
100
1000
10000
r (nm)

B

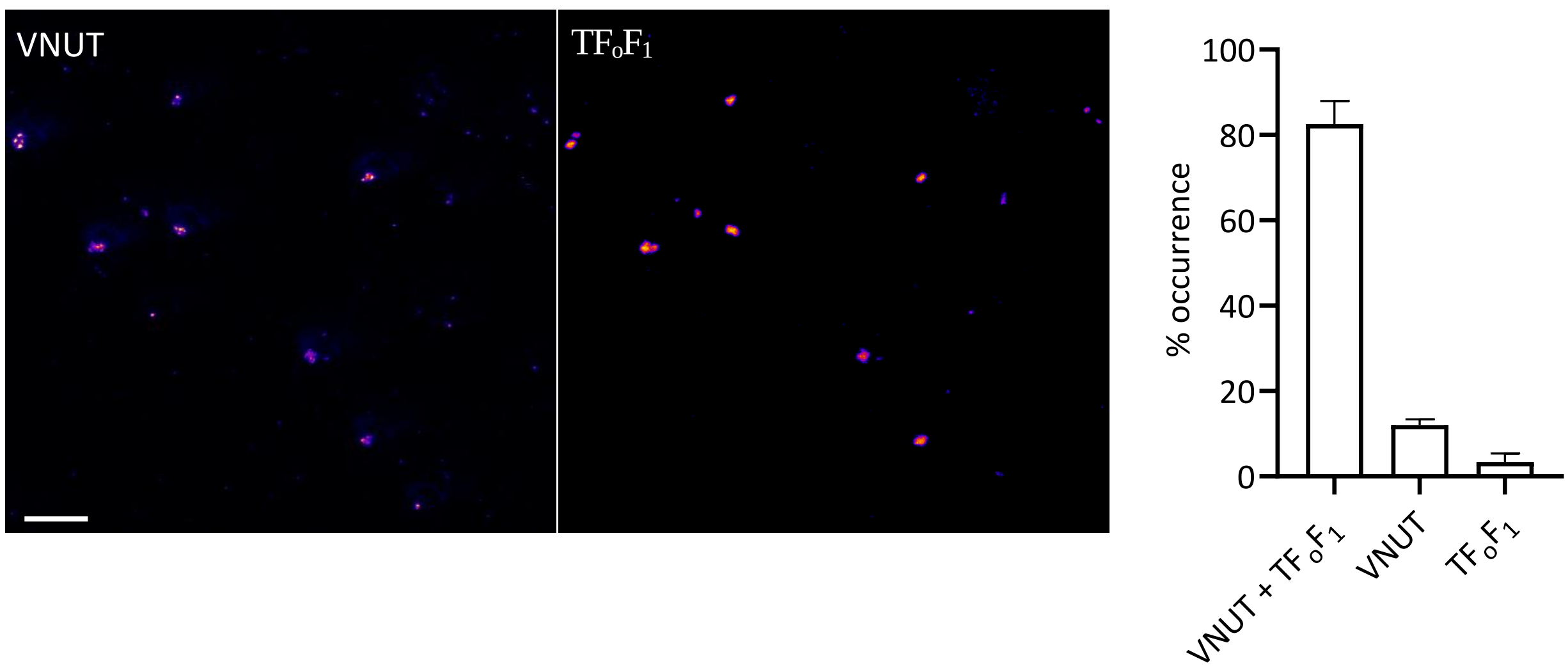

VNUT
TF$_o$F$_1$
% occurrence
100
80
60
40
20
0
VNUT + TF$_o$F$_1$
VNUT
TF$_o$F$_1$

Figure S3

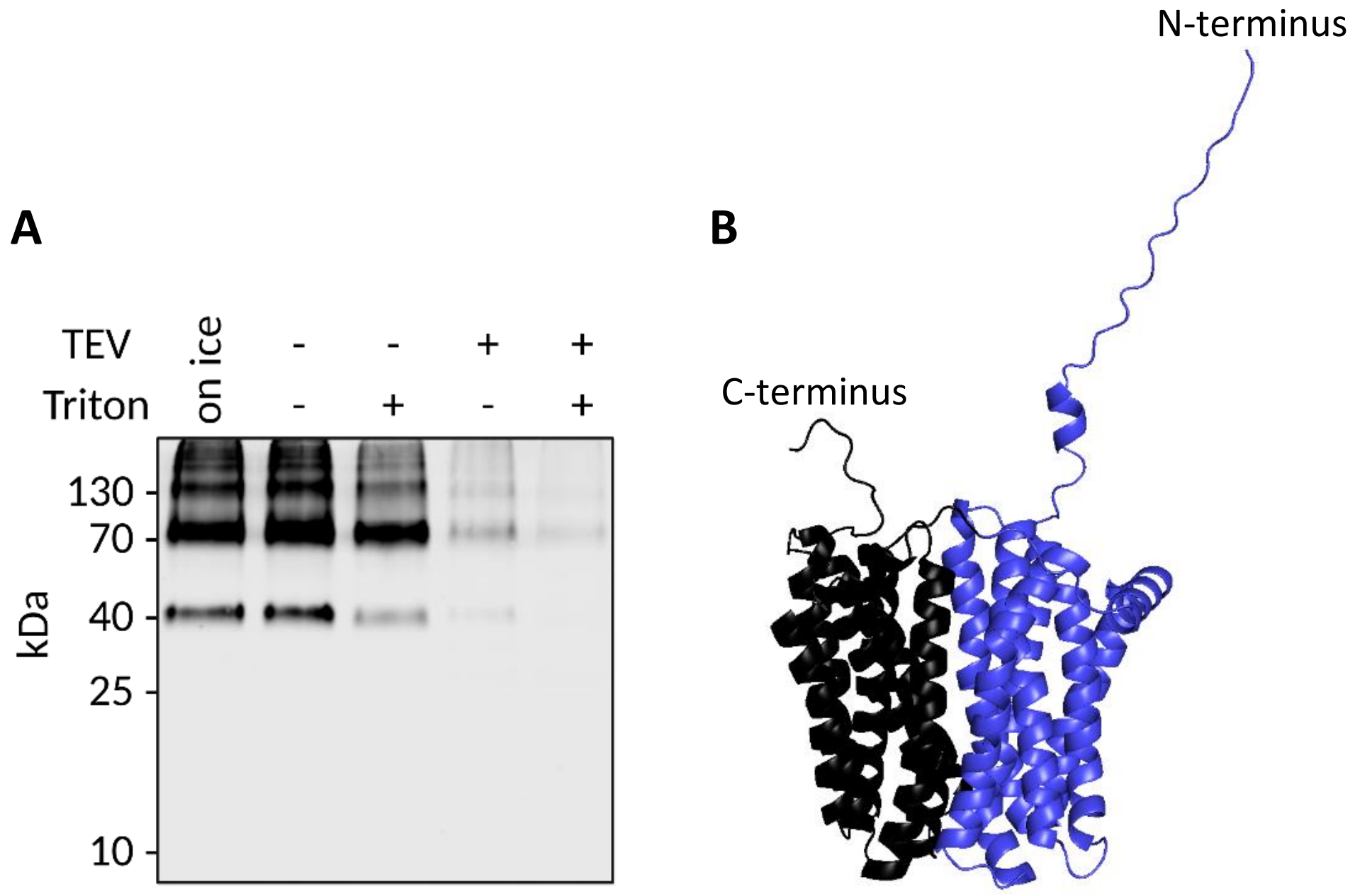

Figure S4

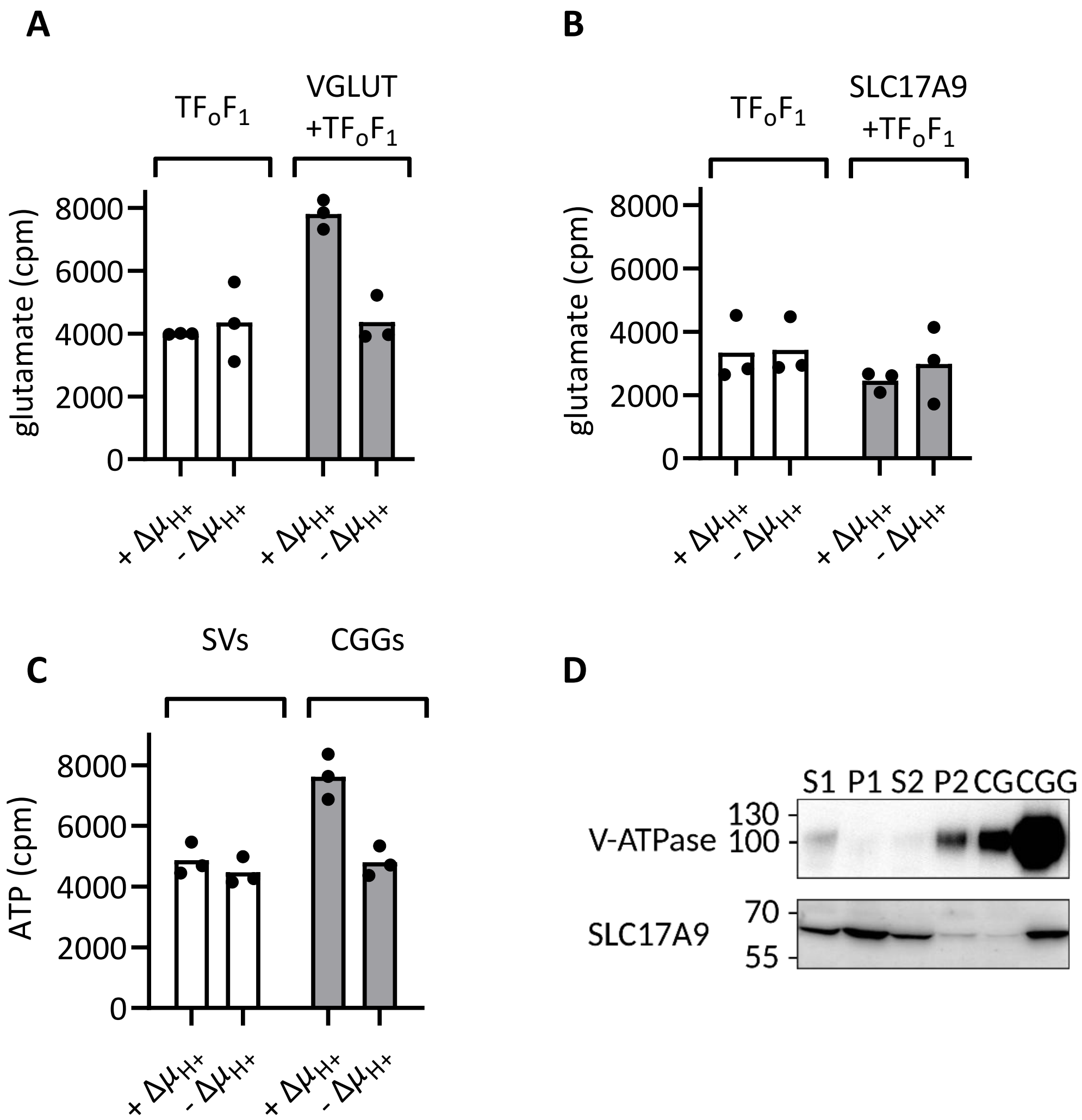
A
TF$_o$F$_1$
VGLUT +TF$_o$F$_1$
glutamate (cpm)
8000
6000
4000
2000
0
+ Δμ$_{H+}$
- Δμ$_{H+}$
B
TF$_o$F$_1$
SLC17A9 +TF$_o$F$_1$
glutamate (cpm)
C
SVs
CGGs
ATP (cpm)
D
S1 P1 S2 P2 CG CGG
V-ATPase
130
100
SLC17A9
70
55
